# An in-Process Inspection System to Detect Noise Originating from within the Interior Trim Panels of Car Doors

**DOI:** 10.3390/s20030630

**Published:** 2020-01-23

**Authors:** Woonsang Baek, Duck Young Kim

**Affiliations:** Department of System Design and Control Engineering, Ulsan National Institute of Science and Technology, Ulsan 44919, Korea; wsbaek@unist.ac.kr

**Keywords:** in-process inspection, acoustic sensor, sound source localization

## Abstract

Car body parts are sometimes responsible for irritating noise caused by assembly defects. Typically, various types of noise are known to originate from within the interior trim panels of car doors. This noise is considered to be an important factor that degrades the emotional satisfaction of the driver of the car. This research suggests an in-process inspection system consisting of an inspection workstation and a noise detection method. The inspection workstation presses down the car door trim panel by using a pneumatic pusher while microphones record the acoustic signals directly above the door trim panel and on the four sides of the workstation. The collected signals are analyzed by the proposed noise detection method after applying noise reduction. The noise detection method determines the presence of irritating noise by using noise source localization in combination with the time difference of arrival method and the relative signal strengths. The performance of the in-process noise detection system was evaluated by conducting experiments on faulty and healthy car door trim panels.

## 1. Introduction

The powertrain, drivetrain, tire contact with the road surface, and climate-control system are considered to be major sources of annoying noise audible in the interior of a car. This noise can adversely affect the driver’s ability to concentrate [1]. Recent advances in noise control technology regarding these systems have successfully reduced the overall level of persistent interior noise [2,3]. However, the decreased noise level paradoxically draws the driver’s attention to weakly audible and occasional interior types of noise known as buzzing, squeaking, and rattling (BSR) noise. Recent research determined the BSR noise in vehicles to be an important cause of customer dissatisfaction [4,5]. Especially, in the case of the car door trim panel, Hyundai Motor and Kia Motors reported that 47% of the recalled car door trim panels in 2013 were claimed to be responsible for BSR noise generated while driving

**Figure 1:**
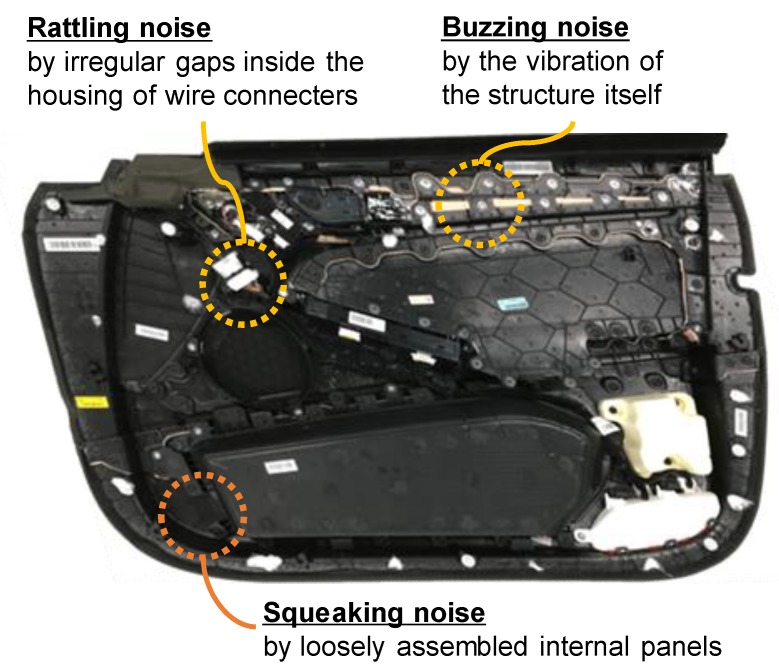
Possible sources of noise in a car door trim panel: buzzing, squeaking, and rattling noise.

The increasing attention paid to BSR noise has led a number of researchers to investigate and study the mechanical reason(s) for the noise. Broadly speaking, BSR noise is defined as noise that varies in the low-frequency band and is caused by movements between loosening or tightening assemblies while driving on the road [6,7]. More specifically, as shown in Figure 1, buzzing is defined as the noise generated by the vibration of the structure itself while driving [8]. Squeaking is the noise emitted by the frictional movement between two body parts of the car, and rattling is the noise generated by the collision between two parts [9]. These types of noise are only determined as BSR if they are sufficiently loud to generate sound-power levels that are audible [10].

Increasing concerns about BSR noise have prompted automakers to pay attention to reduce this type of noise. Table 1 summarizes some inspection methods for BSR noises in industry. The best approach to solve the problem is to identify possible defects in the parts at the early stage of the manufacturing process, especially at the design stage. Recently, a design based on computer aided engineering (CAE) was applied to potentially identify car components that could possibly be responsible for causing BSR noise [10], such as car door seals [11], and instrument panels [12]. However, the highly complex, nonlinear, and uncertain nature of this noise, which could be caused by interactions between any of thousands of parts has thus far challenged the development of a robust detection method with CAE.

Thus, another approach, namely conducting sampling inspection in the laboratory, has been suggested. These inspections are generally conducted by reproducing the bumpy road environment with artificial vibration generators containing shaker-fixtures [13]. Most research utilized microphones to capture the acoustic signals. A number of signal-processing methods have been developed to reproduce the human perception for determining the presence of BSR noise. In general, the presence of BSR noise is not determined by using basic physical quantities such as the sound pressure level due to the low signal-to-noise ratio (SNR). Instead, because the low SNR is the result of contamination by background noise, simple noise reduction methods have been applied.

In this regard, the easiest way to remove the background noise is to filter the collected signal by applying a high-pass filter, which assumes the background noise to be primarily distributed in the low-frequency bands. However, the possibility of making an erroneous decision when removing all the low-frequency bands exists because the frequency components of the actual background noise are usually non-stationary. Thus, considering the non-stationary nature of background noise, methods based on time-frequency characterization such as wavelet transform, were suggested to reduce the accidental elimination of BSR noise [14].

However, laboratory inspection is limited to representative vehicles to shorten the development cycle. Apart from this, sampling inspection methods pose the risk of affecting the quality of products, and therefore it is necessary to develop an efficient in-process inspection system [15,16]. In fact, automakers have been applying the find-and-fix method at the end of the manufacturing process, which inevitably increases the inspection costs [17]. The method is highly subjective and time consuming because the identification and measurement of BSR noise rely on subjective metrics written by human auditors after driving a sample vehicle on a test track [18].

Therefore, the necessity of developing inspection systems capable of conducting an inspection regarding the state of parts during the manufacturing process without affecting the cycle time has increased. This paper presents an in-process BSR noise detection system for car door trim panels. An inspection workstation is designed to reproduce situations in which an inspection engineer presses against the car door trim panel, and acoustic data are collected by two groups of microphones during the inspection. The presence of BSR noise is determined by applying the time difference of arrival and the relative signal strengths of the signals. The results of a practical test involving real car door trim panels provide empirical support for the detection performance of the developed system.

## 2. Proposed in-Process BSR Noise Detection System

**Figure 2:**
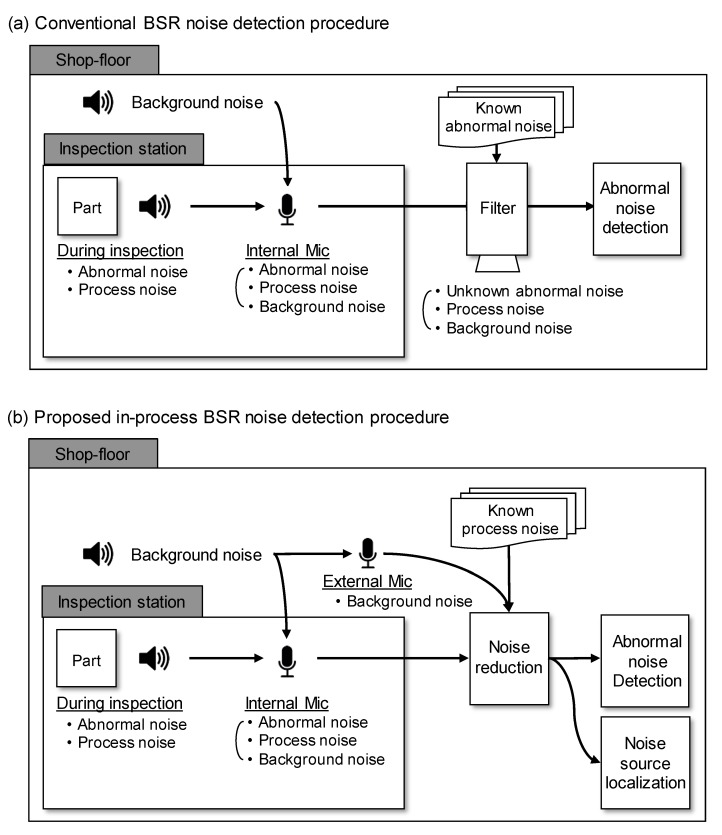
Workflow of BSR noise detection systems: (**a**) conventional and (**b**) proposed in-process BSR noise detection procedures.

The in-process BSR noise detection system consists of an inspection workstation and a noise detection method. The system is designed based on the assumptions about the acoustic signals shown in Figure 2. Acoustic signals generated during the inspection process are categorized into three types of noise, i.e., abnormal noise, process noise, and background noise. Abnormal noise is defined as noise that is generated inside the inspection machine during the inspection process, only when the inspected parts contain an unexpected defect. Process noise is defined as the noise that is always generated by the inspection process of the workstation, for instance the knocking sound generated by the contact between a gantry robot and the parts being tested. Background noise is defined as the daily noise generated in the vicinity of the inspection machine and shop-floor.

The conventional approach of noise detection using microphones is represented as in Figure 2a. In this case, detection is usually conducted using the signals collected by microphones installed inside the inspection workstation. The direct analysis of acoustic signals is difficult because these signals are easily contaminated by background noise. This necessitates the use of a noise reduction procedure, which utilizes subtractive noise reduction after estimation of the background noise [19]. However, because the microphones installed inside the workstation record all types of noise, it is impossible to estimate the exact background noise by using the signal-processing methods. Traditional noise reduction methods utilized trained background noise instead of estimation with signal-processing methods. The possibility of these methods imperfectly reducing the background noise always exists, because the shop-floor environment is characterized by many types of unexpected background noise that cannot be trained.

Therefore, we suggest the in-process BSR noise detection system depicted in Figure 2b, which utilizes two groups of microphones to improve noise reduction and noise detection. In particular, the proposed system supports the performance of the conventional background noise reduction system by providing information about the background noise in real time. In addition, not only does the proposed system aim to achieve noise reduction, it provides an estimated location of the noise-reduced signal by providing geological information of the defective part of the product.

Details of the constructed inspection station and the signal-processing procedure are introduced in subsequent sections.

### 2.1. Configuration of the in-Process BSR Noise Detection System

**Figure 3:**
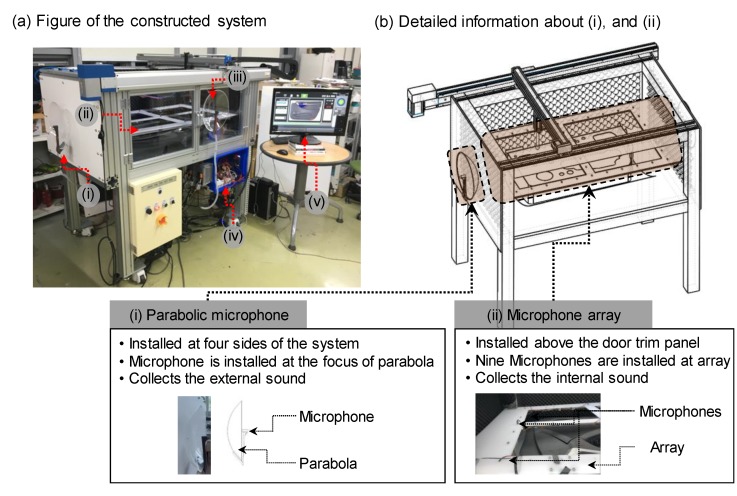
Configuration of the in-process BSR noise detection system: (**a**) image of the constructed system consisting of (i) four parabolic microphones, (ii) a sensor array of nine microphones, (iii) a pneumatic pusher controlled by a gantry robot, (iv) a data acquisition system, and (v) noise detection software; (**b**) illustration showing details about the (i) parabolic microphones and (ii) microphone array. The system detects abnormal noise generated in the defective door trim panel during the inspection process by using the acoustic data captured by the two groups of microphones.

Figure 3 shows the configuration of the developed inspection workstation. The workstation consists of (i) four parabolic microphones, (ii) a sensor array of nine microphones, (iii) a pneumatic pusher controlled by a gantry robot, (iv) a data acquisition system, and (v) noise detection software.

We assumed that the inspection process is conducted at the end of the process in which the car door trim panels are manufactured. The fully assembled car door trim panel is transferred into the workstation and fixed to jigs installed at the bottom. Then, the car door trim panel is slowly pressed down for two seconds by the pneumatic pusher with a pressure of 10 kgf/cm^2^. The acoustic signals are simultaneously captured by the two groups of microphones consisting of the microphone array and the parabolic microphones. The data acquisition system collects the acoustic signals at a sampling rate of 44,100 Hz, which is similar to that used by typical acoustic signal-processing methods. Each group of microphones is designed to concentrate on the respective different types of acoustic signals. The microphones of the microphone array are assumed to collect a mixture of abnormal noise, process noise, and background noise, whereas the parabolic microphones are assumed to intensively collect background noise only.

The microphone array is positioned 50 mm above the car door trim panel to enable it to more effectively capture the acoustic signals of the three different types of nose originating from the panel. The microphones are mounted at each vertex and corner of the acrylic rectangular plate, the size of which is sufficient to cover the car door trim panel. Surplus space between the microphones of the array is removed to allow the pneumatic pusher to operate. Because the number of microphones and the processing time of the noise detection method are proportionally related, it is necessary to determine the appropriate number of microphones. We conducted a simple test to compare the accuracy of noise source localization and processing time because the localization process is the most time-consuming step of the noise detection method.

The space between the microphones is divided into uniform rectangles according to the arrangement of microphones. The precision of localization is determined by estimating the position at which noise is generated 10 times and by using 6, 9, and 12 microphones. As expected, the processing time increased sharply with the number of microphones, with the detection precision of 9 and 12 microphones being similar, whereas that obtained with 6 microphones was relatively low. Thus, 9 microphones were selected and installed as the microphone array.

On the other hand, four parabolic microphones were installed in parallel at the center and pointing outward to the four corners of the system to capture the shop-floor background noise more effectively, while being insensitive to the sounds emitted inside the workstation during the inspection process. Each parabola is designed to have a holder to which to attach the microphone at its focus. Note that a parabolic microphone is useful to attenuate the noise originating from the back of the parabola [20].

**Figure 4:**
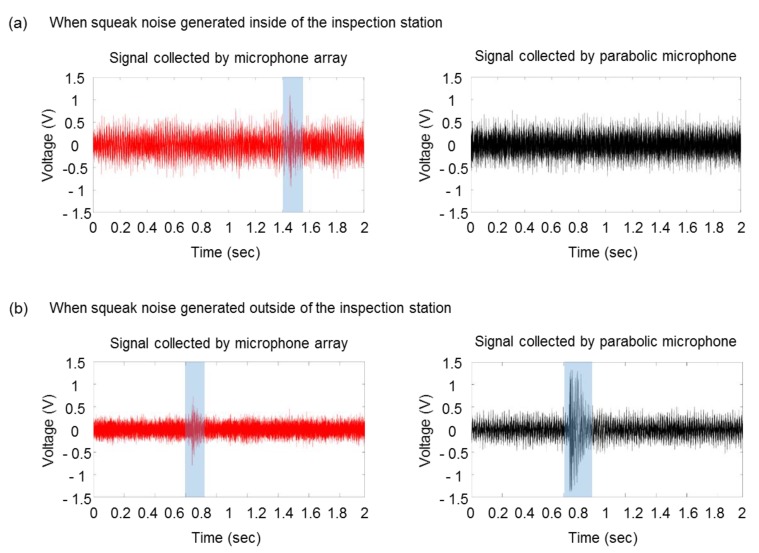
Different responses of microphone array and parabolic microphone when squeak noise is generated (**a**) inside of the inspection station and (**b**) outside of the inspection station. (**a**) A spark near 1.4 s is only found in signal of the microphone array. (**b**) A spark near 0.8 s can be found in both groups of microphones.

Figure 4 shows the different responses of a microphone of the microphone array and a parabolic microphone when squeaking noise is generated inside and outside the inspection station. When the noise is generated inside of the inspection station, the signal collected by the microphone array is only that generated by squeaking noise. On the other hand, squeaking noise can be found near 0.8 s in both groups of microphones when the noise is generated outside the inspection station. Then the parabolic microphones can be assumed to mainly collect background noise, whereas the microphone array is assumed to collect all three types of noise in situations in which the background noise is attenuated.

### 2.2. In-Process BSR Noise Detection Procedure

#### 2.2.1. Data Acquisition

**Figure 5:**
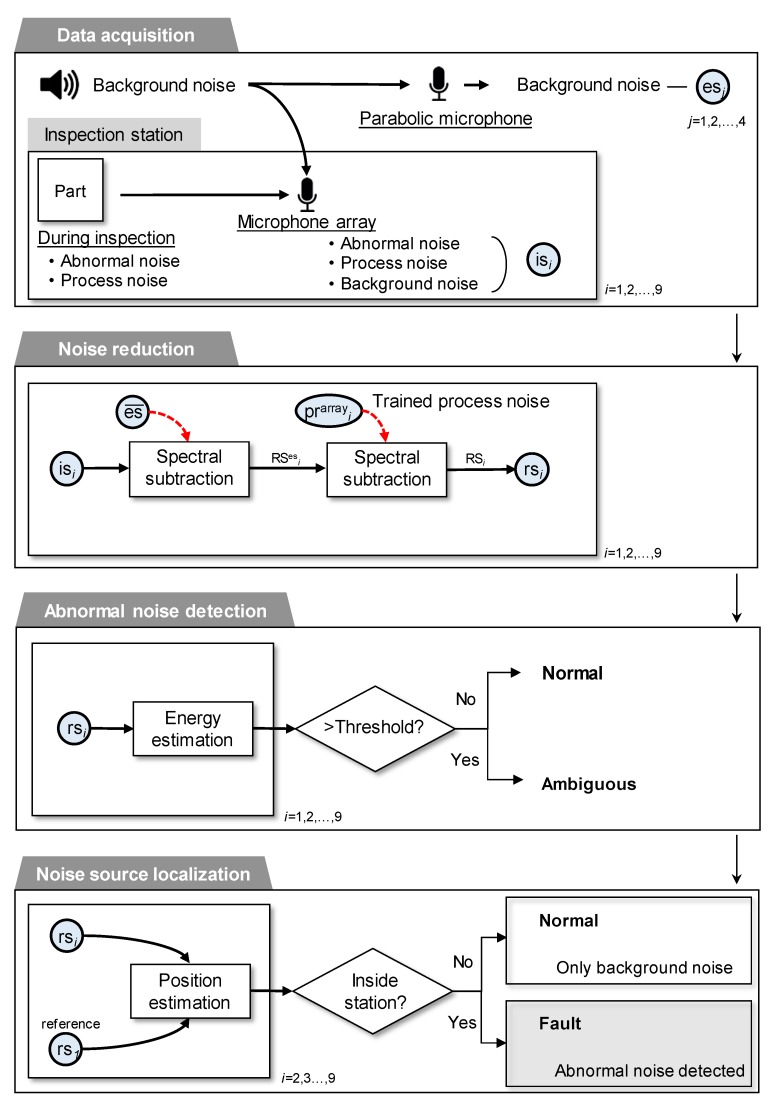
Overview of the four phases of the in-process BSR detection procedure: Data acquisition, Noise reduction, abnormal noise detection, and Noise source localization.

The proposed in-process BSR detection procedure consists of four phases as shown in Figure 5: (i) data acquisition, (ii) noise reduction, (iii) abnormal noise detection, and (iv) noise source localization.

As mentioned before, the signals recorded by the microphones are categorized as originating from abnormal, process, and background noise. Specifically, the abnormal, process, and background noise collected by the *i*th microphone in the microphone array are defined as an^array^*_i_*[n], pr^array^*_i_*[n], and bn^array^*_i_*[n], respectively, where *i* = 1, 2, …, 9 and *n* = 1, 2…, 200. The noise collected by the *j*th parabolic microphone is defined as an^para^*_j_*[*n*], bn^para^*_j_*[*n*], and pr^para^*_j_*[*n*], respectively. The signal collected by the *i*th microphone in the microphone array is defined as is*_i_*[*n*], and the signal collected by the *j*th parabolic microphone is defined as es*_j_*[*n*]. Assuming that the three types of noise are statistically independent, is*_i_*[*n*] and es*_i_*[*n*] can be represented as below:

is*_i_*[*n*] = an^array^*_i_*[*n*] + bn^array^*_i_*[*n*] + pr^array^*_i_*[*n*] (1)

es*_j_*[*n*] = an^para^*_j_*[*n*] + bn^para^*_j_*[*n*] + pr^para^*_j_*[*n*] (2)

Based on the nature of omnidirectional recording of parabolic microphones described in the previous section, the magnitudes of anparaj[*n*] and pr^para^*_j_*[*n*] are considered to be negligibly small in es*_j_*[*n*]. Then, we can rewrite (2) as below:

(3) $${\mathrm{es}}_{j}[n]\;≅\;{{\mathrm{bn}}^{\mathrm{para}}}_{j}[n]$$

The process noise, pr^array^*_i_*[*n*], is defined as the noise generated during operation of the pneumatic pusher and the actuators for inspection and pr^array^*_i_*[*n*] is the average of the signals collected by repeating the inspection 150 times with healthy car door trim panels. Because the collected signals are considered to contain both process noise and background noise, pr^array^*_i_*[*n*] is obtained by applying noise reduction to the collected signal.

#### 2.2.2. Noise Reduction

The objective of the noise reduction step is to highlight an^array^*_i_*[*n*] from is*_i_*[*n*] by removing pr^array^*_i_*[*n*] and es*_j_*[*n*] as shown in Figure 5. First, is*_i_*[*n*] and esj[n] are represented in the time-frequency domain by applying short time Fourier transform (STFT), IS*_i_*[*w*,*m*] and ES*_j_*[*w*,*m*], where the window size *w* is determined as 172 and the size of the frequency bin m as 258. The time-frequency information of es*_j_*[*n*] is removed from IS*_i_*[*w*,*m*] by applying a spectral subtraction method [21,22,23], namely multiplying the original spectrum by its attenuation factor AF^es^*_i_*[*w*,*m*], which yields the spectrum of the remaining signal RS^es^*_i_*[*w*,*m*]:

RS^es^*_i_*[*w*,*m*] = IS*_i_*[*w*,*m*]◌AF^es^*_i_*[*w*,*m*] (4)

where ◌ represents element-wise multiplication between two spectra. Likewise, the final spectrum of the remaining signal RS*_i_*[*w*,*m*] is obtained by removing the time-frequency information of pr^array^*_i_*[*n*] from RS^es^*_i_*[*w*,*m*].

The attenuation factor is generally obtained by averaging the time-frequency components of trained background noise. Some variants of spectral subtraction methods proposed over-estimation of the noise spectrum and spectral flooring for non-stationary noises [22,24]. Here, the empirical Wiener attenuation rule is applied to set the attenuation factor because it minimizes the sum of the distortion of signals and energy [25].

(5) $${{\mathrm{AF}}^{\mathrm{es}}}_{i}[w,m]\;=\;{\left(1-\lambda {\left[\frac{1}{\xi_{i}^{\mathrm{es}}\left[w,m\right]+1}\right]}^{\beta_{1}}\right)}^{\beta_{2}}$$

where $\beta_{1},\;\beta_{2}\;\geq \;0$ and λ ≥ 1 are the over-subtraction factors and $\xi_{i}^{\mathrm{es}}\left[w,m\right]$ is the SNR of the power spectrum. The soft-thresholding method is applied to determine the over-subtraction factors [22,26]; hence, the following values are specified: $\beta_{1}\;=\;0.5{,\;\beta }_{2}\;=\;1$, and λ = 10. $\xi_{i}^{\mathrm{es}}\left[w,m\right]$ is then represented as follows:

(6) $$\xi_{i}^{\mathrm{es}}\left[w,m\right]\;=\;\frac{{\mathrm{IS}}_{i}{\left[w,m\right]}^{2}}{\mathrm{ES}{\left[w,m\right]}^{2}}$$

where ES[w,m] is obtained by averaging all ${\mathrm{ES}}_{j}\left[w,m\right]$, where *j* = 1, 2, …, 4. The remaining signal RS^es^*_i_*[*w*,*m*] computed by using (4) is assumed to contain the abnormal noise and process noise. Subsequently, spectral subtraction is applied to RS^es^*_i_*[*w*,*m*] to remove the spectrum of the process noise, PR^array^*_i_*[*w*,*m*]. To do this, the attenuation factor for the spectral subtraction of ${{\mathrm{AF}}^{\mathrm{pr}}}_{i}\left[w,m\right]$ is defined as follows:

(7) $${{\mathrm{AF}}^{\mathrm{pr}}}_{i}[w,m]\;=\;{\left(1-\lambda {\left[\frac{1}{\xi_{i}^{\mathrm{pr}}\left[w,m\right]+1}\right]}^{\beta_{1}}\right)}^{\beta_{2}}\;\;$$

where $\xi^{\mathrm{pr}}{}_{i}\left[w,m\right]$ is defined as follows:

(8) $$\xi_{i}^{\mathrm{pr}}\left[w,m\right]\;=\;\frac{{\mathrm{RS}}_{i}^{\mathrm{es}}{\left[w,m\right]}^{2}}{{\mathrm{PR}}_{i}{\left[w,m\right]}^{2}}\;\;$$

Finally, the spectrum of the remaining signal, RSi[w,m], is obtained as follows:

(9) $${\mathrm{RS}}_{i}[w,m]\;=\;{{\mathrm{RS}}^{\mathrm{es}}}_{i}[w,m]◌{{\mathrm{AF}}^{\mathrm{pr}}}_{i}\left[w,m\right]\;\;$$

and the remaining signal rs*_i_*[*n*] is obtained by applying inverse STFT to RS*_i_*[*w*,*m*].

#### 2.2.3. Abnormal Noise Detection

The abnormal noise detection procedure is conducted to verify whether any remaining noise is present in rs*_i_*[*n*]. After applying noise reduction, the remaining signal, rs*_i_*[*n*] is assumed to contain abnormal noise only when the car door trim panel is faulty. Alternatively, rs*_i_*[*n*] is assumed to be null when the car door trim panel is healthy. The existence of any abnormal noise in rs*_i_*[*n*] is determined by comparing a measure of the signal in the form of the energy of the signal with the predefined threshold [27,28].

(10) $${\mathrm{energy}}_{i}\;=\;\overset{W}{\underset{w=1}{\sum }}\overset{M}{\underset{m\;=\;1}{\sum }}{\left|{\mathrm{RS}}_{i}[w,m]\right|}^{2}$$

The reference energy as a threshold is obtained by averaging the energy of the noise-reduced signals that were recorded by repeating the inspections 150 times with healthy car door trim panels. In other words, the reference energies are assumed to have no significant power because the background noise and the process noise are removed. The reference energy of the nine microphones is compared with the energy of rs*_i_*[*n*]. If the energy of any rs*_i_*[*n*] is significantly larger than the reference energy, it leads to conclude that abnormal noise may exist.

#### 2.2.4. Noise Source Localization

There is a possibility that, if the energy of rs*_i_*[*n*] exceeds that of the threshold, then the remaining noise may contain background noise that has not been perfectly removed because of the limitation of the noise reduction method. Thus, determining the existence of abnormal noise in the remaining signal by only comparing the energy could lead to an ambiguous result. This problem was solved by considering the estimated location of the dominant signal in rs*_i_*[*n*] as supplementary information. If the dominant signal is estimated to exist inside the inspection workstation, abnormal noise is assumed to exist in rs*_i_*[*n*]. In the opposite case, rs*_i_*[*n*] is assumed to predominantly comprise residual background noise [29].

Localization of the dominant signal in rs*_i_*[*n*] is conducted by applying the hyperbolic time difference of arrivals (TDOA) method [30,31,32]. The method utilizes the derived arrival delay time and the geological location of pairs of microphones for localization. The generalized cross-correlation with phase transform method (GCC-PHAT), which is useful to calculate the cross-correlation of noisy data, is employed for the efficient computation of the arrival delay time [33].

The *i*th cross correlation, gcc*_i_*[*n*], is defined as the cross correlation between a signal of a fixed microphone and the *i*th microphone, rs_1_[*n*] and rs*_i_*[*n*], respectively, where *i* = 2, 3, …, 9. Specifically, gcc*_i_*[*n*] is obtained by applying inverse fast Fourier transform (IFFT) to the multiplication of the weighting function and the cross-spectral density between rs_1_[*n*], and rs*_i_*[*n*] as follows [34]:

(11) $${\mathrm{gcc}}_{i}[n]\;=\;F^{-1}\left(\frac{F[{\mathrm{rs}}_{1}]\;F{\left[{\mathrm{rs}}_{i}\right]}^{*}}{\left|F[{\mathrm{rs}}_{1}]\;F{\left[{\mathrm{rs}}_{i}\right]}^{*}\right|}\right)$$

where F[${\mathrm{rs}}_{1}$] is the Fourier transform of ${\mathrm{rs}}_{i}$, * denotes the complex conjugate, and *k* = 2, 3, …, 9. Then, the arrival delay time between two signals, $\tau_{i}$, is estimated by finding the maximum peak among ${\mathrm{gcc}}_{i}\left[n\right]$ as follows:

(12) $$\tau_{i}\;=\;\underset{\;n}{\arg\;\max\;}{\mathrm{gcc}}_{i}\left[n\right]$$

**Figure 6:**
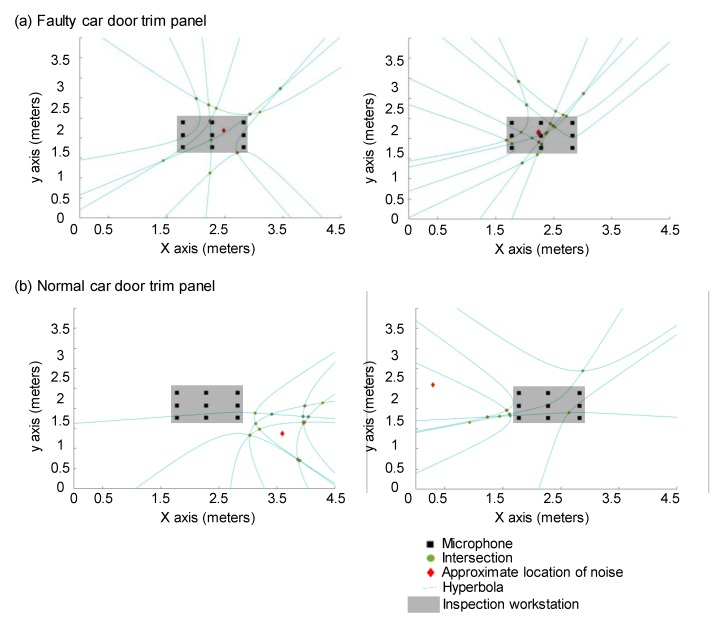
Examples of noise source localization. (**a**) Analysis of data of faulty car door trim: estimated location of noise is the inside of the inspection workstation. (**b**) Analysis of data of healthy car door trim: estimated location of noise is the outside of the inspection workstation.

Then, eight hyperbolas are obtained by considering the locations of each microphone as loci. The dominant signal is assumed to lie on one of the two branches of each hyperbola [35]. The intersections between the hyperbolas are assumed to be the possible location of the dominant noise. By computing the middle points of the intersections between all the hyperbolas, we finally estimate the location of the dominant signal. If the location is estimated to be inside the rectangle formed by the microphone array, the car door trim panel is determined to be faulty. Otherwise, the car door trim panel is determined to be healthy. Figure 6 shows examples of noise source localization using signals captured during the inspection of car door trim panels that are both faulty and healthy. When an analysis of the remaining signals of faulty car door trim shows that the estimated locations are inside the inspection workstation, then the car door trim panel is determined to be faulty. Alternatively, when an analysis of the remaining signals of faulty car door trim shows that the estimated locations are outside the inspection workstation, the car door trim panels are determined to be healthy.

## 3. Experimental Results and Discussion

**Figure 7:**
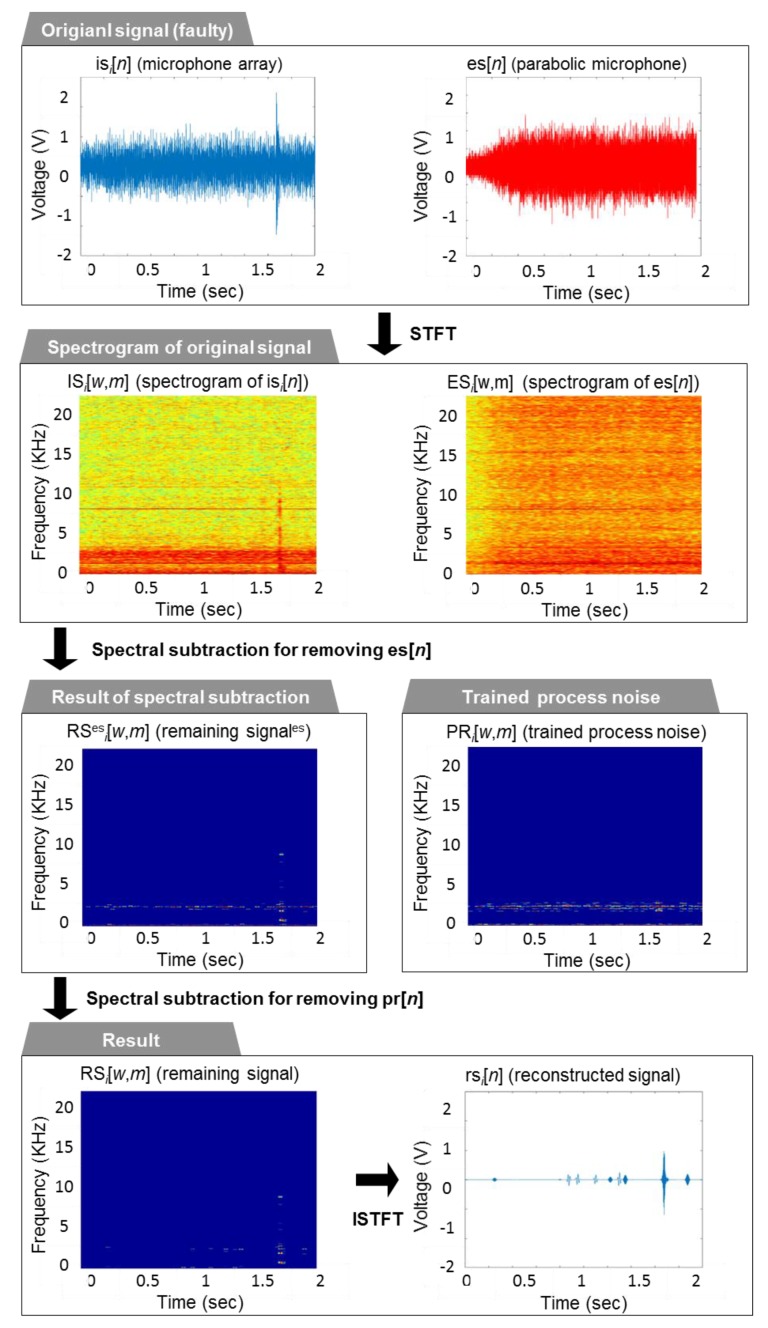
Noise reduction in the recorded acoustic signal from a faulty door trim panel.

We conducted BSR noise inspection experiments with two types of car door trim panels: two faulty door trim panels which were known to emit BSR noise and two healthy door trim panels. The inspection was repeated 100 times for each type of door trim panel (i.e., 50 repetitions for each panel). The acoustic signals were recorded for two seconds right after the pneumatic pusher starts pressing down the door trim panel. Figure 7 illustrates the procedure of noise reduction in the recorded acoustic signal from a faulty door trim panel.

**Figure 8:**
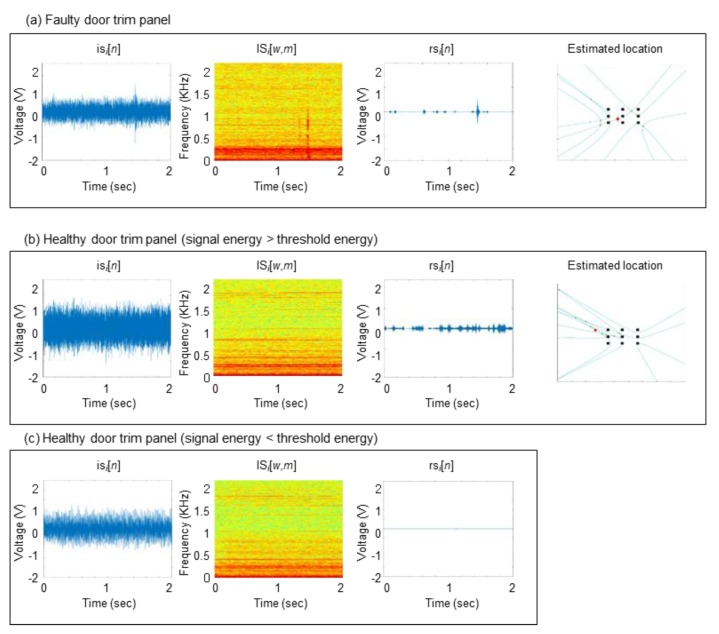
Three examples of noise inspection results: (**a**) correct determination of a faulty car door trim panel (**b**) correct determination of a healthy panel when the energy of rs*_i_*[*n*] exceeds the threshold energy (**c**) correct determination of a healthy panel.

Figure 8 shows three noise detection cases selected from the inspection results. First, in the case of a faulty door trim panel as shown in Figure 8a, the energy of rs*_i_*[*n*] exceeds the predefined threshold, and further it is estimated that the noise is coming from inside the workstation. As a result, we can conclude that the panel is generating noise. Second, in cases of a healthy door trim panel as shown in Figure 8b, the energy of rs*_i_*[*n*] can exceed the predefined threshold due to irregular background noise. However, by noise source localization, we can estimate that the noise is coming only from outside the workstation, and consequently we can conclude that the panel is healthy. Last, if the energy of rs*_i_*[*n*] does not exceed the predefined threshold as shown in Figure 8c, we can conclude that the panel is healthy.

We compared the performance of the proposed method with a popular noise reduction method using the high-pass filter. Note that the high-pass filter, by its nature, removed the pre-specified low-frequency components, assuming them as the background noise. The pass band of the filter was determined with empirical experiments and was set to be 500 Hz. As summarized in Table 2 and Table 3, the percentage of correct decision rates for the faulty and healthy door trim panels are 99% and 96% for the proposed method, and 85% and 90% for the conventional method, respectively. Nonetheless, the number of incorrect decisions for the proposed method is not negligible. Clearly, the correct decision rate of healthy car door trim panels is considerably lower than that of the faulty car door trim panels. First, this could be caused by the methodological limitations of spectral subtraction of the noise reduction stage. The remaining signal of healthy car door trim panels may have strong musical noise which is greater than the threshold.

As mentioned earlier, the performance of the spectral subtraction highly depends on the parameters, and cannot process situations in which the spectrum of the background noise overlaps with the spectrum of abnormal noise. This problem could be solved by applying a machine-learning technique to spectral subtraction to reduce the musical noise. Alternatively, it would be necessary to develop a novel noise reduction method that assumes statistical dependence between the different types of noise.

Regarding the errors generated during the noise source localization step, the arrival times may not be obtained accurately due to errors in the noise reduction. A problem would also arise when only a few of the microphones detected a signal that is not shared with all the others, in which case it could also reduce the accuracy of localization.

In addition, abnormal detection utilizes only the energy of a signal, which does not consider the time-varying characteristics of the acoustic signals. A new detection method based on the pattern recognition technique would enable the types of BSR noise to be distinguished. The detection precision would also be increased because the patterns would be able to consider various characteristics of signals. This would not only improve the effectiveness of the algorithms, but also the configuration of the workstation.

The acoustic signals are highly affected by the data collection environment, such as the direction of each microphone, and the presence of obstacles that generally cause signals to reverberate. The proposed methods could be used to assess the arrangement of the microphones and different shapes of the microphone array. We aim to implement these improvements in future studies by using empirical experiments.

## 4. Conclusions

This study presented an in-process BSR noise detection system for car door trims. The system was developed for overcoming the limitations of the conventional BSR noise detection methods, such as model based pre-evaluation inspection, test-drive based pre-evaluation, and sampling inspection. To do so, the system installed the two groups of microphones and applied the noise reduction method.

A signal analysis method that performs noise reduction, abnormal noise detection, and noise localization was applied to determine whether abnormal noise is present. In particular, the method utilizes a spectral subtraction method, hyperbolic TDOA approach, and compares the energy of signals. A total of 200 BSR inspection results with real car door trim panels demonstrated 99% accuracy for faulty car door trim panels, and 96% for healthy car door trim panels. The performance comparison with the high-pass filter-based method showed that the proposed method efficiently reduced the background noise while preserving the signal of interest.

For further study, it is necessary to consider the spectral overlap between the signal of interest and the background noise. In other words, various sound sources must be efficiently separated to extract the frequency components of the signal of interest. Moreover, signal energy evaluation for noise detection decision can be further improved by applying pattern recognition techniques.

## Figures and Tables

**Table 1:** Comparison of the BSR noise inspection methods.

Method	Description
Model based pre-evaluation [10,11,12]	Expects the existence of the BSR noise using the finite element analysis Can describe the mechanism of BSR noise generation in a body structure
Test-drive based pre-evaluation [17,18]	Manually evaluate vehicles on the test track to measure the noise level Can estimate the noise level that the actual customers would experience The result is highly subjective and the procedure is time consuming
Sampling inspection [13,14]	Examines the existence of the BSR noise by using various test methods in an anechoic chamber Can objectively determine the existence of the BSR noise with the sensors and testing methods The test procedure is very expensive in time and cost

**Table 2:** The experimental results of the 200 inspections (conventional method).

Dataset	Decision				
	Hit	False Alarm	Miss	Correct Rejection	Correct Decision Rate
**Faulty Door Trim**	85	0	15	0	85%
**Normal Door Trim**	0	10	0	90	90%

**Table 3:** The experimental results of the 200 inspections (proposed method).

Dataset	Decision				
	Hit	False Alarm	Miss	Correct Rejection	Correct Decision Rate
**Faulty Door Trim**	99	0	1	0	99%
**Normal Door Trim**	0	4	0	96	96%
